# Whole genome doubling-induced the enrichment of H3K27me3 in genes carrying specific TEs in *Aegilops tauschii*

**DOI:** 10.3389/fgene.2023.1241201

**Published:** 2023-07-25

**Authors:** Hongwei Guo, Guoyan Zhang, Min Zhou, Min Wan, Bo Zhu, Zujun Yang, Deying Zeng, Zixian Zeng

**Affiliations:** ^1^ Department of Biological Science, College of Life Sciences, Sichuan Normal University, Chengdu, Sichuan, China; ^2^ Horticulture Institute, Sichuan Academy of Agricultural Sciences, Chengdu, China; ^3^ Plant Functional Genomics and Bioinformatics Research Center, Sichuan Normal University, Chengdu, Sichuan, China; ^4^ Center for Informational Biology, School of Life Science and Technology, University of Electronic Science and Technology of China, Chengdu, China

**Keywords:** whole-genome doubling, *Aegilops tauschii*, transcriptome, transposable elements, H3K27me3

## Abstract

Polyploidization plays important roles in the evolution and breeding of the common wheat. *Aegilops tauschii*, the D-genome progenitor of the common wheat, provides a valuable pool of resistance genes to multiple diseases. Extensive studies focus on the exploration of these genes for wheat improvement. However, few studies have unveiled alternations on genome-wide expression pattern and histone modifications induced by whole-genome doubling (WGD) process. In this study, we conducted transcriptome analysis for the diploid and tetraploid *Ae. taushcii* lines using the leaf and root tissues. Both lines tend to display similar tissue-specific pattern. Interestingly, we found that TEs located in genic regions were depleted of the repressive histone mark H3K27me3, whereas their adjacent chromatin was enriched with H3K27me3. The tetraploid line exhibited higher levels of H3K27me3 in those regions than the diploid line, particularly for genic regions associated with TEs of the long interspersed nuclear elements (LINEs), CACTA, PIF/Harbinger, Tc1/Mariner and unclassed DNA transposon. Surprisingly, the expression levels of these TEs cognate genes were negatively associated with the levels of H3K27me3 between the tetraploid and diploid lines, suggesting the five types of TEs located within genic regions might be involved in the regulation of the ploidy-related gene expression, possibly through differential enrichment of H3K27me3 in the genic regions. These findings will help to understand the potential role of specific types of TEs on transcription in response to WGD.

## 1 Introduction

Autopolyploids and allopolyploids are considered to be arose from the WGD within a single species and through the merging of genomes from difference species followed by doubling, respectively (Parisod et al., 2010a; Van de Peer et al., 2017). A considerable number of allopolyploid plant species have been investigated, including hexaploid bread wheat (Zhang et al., 2014), *Brassica juncea* (Yang et al., 2016), cotton (Chen Z. J. et al., 2020) and strawberry (Edger et al., 2019). However, studies on autopolyploid plant species are limited to few species (Song et al., 2020; Wang et al., 2021; Guo et al., 2023).

WGD-induced gene expression changes were relatively mild, limited to a few hundred genes in tetraploid potato (Guo et al., 2023) and *Arabidopsis thaliana* (Yu et al., 2010), although the tetraploid derivatives from both species exhibited typical changes in phenotypes, including enlarged tissues. WGD-induced variation in gene expression pattern is affected by multiple factors, such as ploidy level (Auger et al., 2005), DNA sequence alternation (Chen Z. J. et al., 2020), histone modification (Guo et al., 2023), DNA methylation (Wang et al., 2021), as well as TE status (Gill et al., 2021). TEs take effect on the expression of their adjacent genes by various ways, including transposition and production of short interfering RNAs (siRNA) (Wang et al., 2013; Gill et al., 2021). Insertion of TE into genic region may result in aberrant or novel transcripts, while transposition of TE into promoter may disrupt the function of promoter or produce alternative promoter leading to new expression pattern (Hirsch and Springer, 2017). In addition, studies on epigenetic modifications associated with TEs mainly focused on DNA methylation (Madlung et al., 2005; Parisod et al., 2009; Zhang et al., 2015). However, the association of TEs in genic regions with WGD-induced enrichment of histone modifications in their cognate genes, as well as the resulting differential gene expression between ploidies remain unraveled.

*Ae. tauschii* (2n = 2x = 14, DD) is the D-genome progenitor of the hexaploid wheat (2n = 6x = 42, AABBDD) (Van Slageren, 1994) and its genome contains a large number of TEs (84.4%) (Luo et al., 2017). As a wild species, *Ae. tauschii* serves as an important germplasm for wheat improvement, since it contains a number of novel genes resistant to multiple diseases (Cox et al., 2017; Gaurav et al., 2022), such as rust (Lin et al., 2022) and power mildew (Xue et al., 2022). In addition, the synthetic tetraploid *Ae. tauschii* will also be useful as a bridge to generate hybrids or amphiploids by crossing with other tetraploid Triticeae species for wheat breeding (Wang et al., 2010; Mizuno et al., 2011). Thus, investigation of gene expression patterns associated with ploidy in *Ae. tauschii* may provide clues for breeding targets. In this study, we utilized previously developed diploid and tetraploid *Ae. tauschii* and conducted transcriptomes for both leaf and root tissues for each ploidy. Both diploid and tetraploid lines tend to display similar tissue-specific pattern. Interestingly, TEs located in the genic regions were depleted of H3K27me3, whereas their adjacent chromatin was enriched with H3K27me3. The tetraploid line displayed higher levels of H3K27me3 in those regions than the diploid line*.* In particular, we found that the genic regions associated with five types of TEs showed significantly higher levels of H3K27me3 in the tetraploid line compared with those in the diploid line. Surprisingly, the expression levels of these TEs cognate genes were negatively associated with the levels of H3K27me3 between the tetraploid and diploid lines, suggesting the five types of TEs located within genic regions might be involved in the regulation of the ploidy-related gene expression, possibly through differential enrichment of H3K27me3 in the genic regions.

## 2 Materials and methods

### 2.1 Plant materials

The diploid (2n = 2x = 14) and tetraploid (2n = 4x = 28) *Ae. Tauschii*, provided by Professor Huaren Jiang, were grown in a walk-in growth chamber under the photoperiod of 16 h at 24°C daylight and 8 h at 22°C darkness. Both leaves and roots were harvested 60 days after germination, respectively, which were subject to RNA-seq and ChIP-seq experiments or immediately frozen in liquid nitrogen.

### 2.2 RNA-seq and ChIP-seq

Both leaves and roots were used for RNA-seq and ChIP-seq, respectively. Each tissue from three individual plants were pooled together as a biological replicate for each line. Two biological replicates of RNA-seq libraries were developed and sequenced using an Illumina NovaSeq 6000 platform with the mode of 150 pair end (PE) sequencing. All RNA-seq libraries were developed and sequenced in Novagene company.

According to the published protocol (Zhang et al., 2012), the ChIP experiments were performed with the antibody against H3K27me3 (Millipore 07–449). Chromatin was digested into monomer nucleosome pattern (∼150 bp fragments) using MNase (Sigma N3755) to obtain the highest resolution of the histone modification signal. Chromatin in monomer nucleosome pattern carrying H3K27me3 were captured and precipitated using rProtein A Sepharose beads (GE 17-1279-01), followed by ChIP-DNA separation. The isolated ChIP-DNA was applied for library construction, which was subsequently sequenced using the same method as the RNA-seq libraries in Novagene company.

### 2.3 Data analysis

Raw reads generated from RNA-seq and ChIP-seq were first processed for quality control and adapter trimming using the program fsatp v0.32.2 with “-w 8” (Chen et al., 2018). Clean RNA-seq reads were mapped to the *Ae. tauschii* genome assembly (Ensembl v4.0) (Luo et al., 2017), using Hisat2 (Kim et al., 2019). Reads with mapping quality greater than 50 were retained for further analysis. The expression of all annotated genes was called using StringTie v2.1.5 (Pertea et al., 2015) and the differentially expression genes were identified using DESeq2 v1.32.0 (Love et al., 2014) with FDR <0.01, log_2_(Fold Change) > 1. The g:Profiler program (https://biit.cs.ut.ee/gprofiler/gost) was used for the gene ontology enrichment analysis. The statistical test for the reproducibility of RNA-seq data between biological replicates was conducted using Pearson correlation.

Clean ChIP-seq reads were mapped to the *Ae. tauschii* genome assembly, using the program of BWA “mem” with default parameters. The mapped reads were further filtered with mapping quality greater than 50. The uniquely mapped reads (mapped to a unique genomic position) were obtained and processed for downstream analysis. The histone modification signal was defined as the mid-point of the uniquely mapped paired reads. Quantification of the level of a histone modification within an interval was conducted by summarizing histone modification signals and normalizing to length of the interval, PE read number per million uniquely mapped reads and IgG. A histone modification enriched region was identified using the program MACS2 (Zhang et al., 2008) with -q 0.05. Statistical significance was tested using Wilcoxon signed-rank test with paired samples.

### 2.4 Repetitive sequence analysis

Repetitive sequences of the *Ae. tauschii* genome were downloaded from http://aegilops.wheat.ucdavis.edu/ATGSP/annotation/. Genomic distribution of TEs relative to the annotated genes was determined if more than half length of TEs is overlapped with a genomic feature.

### 2.5 Data visualization

All data were visualized using R program (https://www.r-project.org).

## 3 Results

### 3.1 Tissue-specific expression pattern in two ploidies

The tetraploid *Ae. tauschii* line (2n = 4x = 28, DDDD) was previously regenerated from the diploid *Ae. tauschii* (Zeng et al., 2012), one of the progenitors of the common wheat. The tetraploid line was associated with no visible structural variations on the chromosome level (Zeng et al., 2012), suggesting its genome tends to be homozygous at certain degree. However, typical phenotypic polymorphisms, which are frequently identified in other plant species (Miller et al., 2012; Saminathan et al., 2015), were found between two ploidies, including larger but less leaves (Supplementary Figure S1), as well as larger seeds (Zeng et al., 2012). Thus, we utilized the tetraploid derivative and its diploid progenitor for the transcriptome analysis.

**FIGURE 1:**
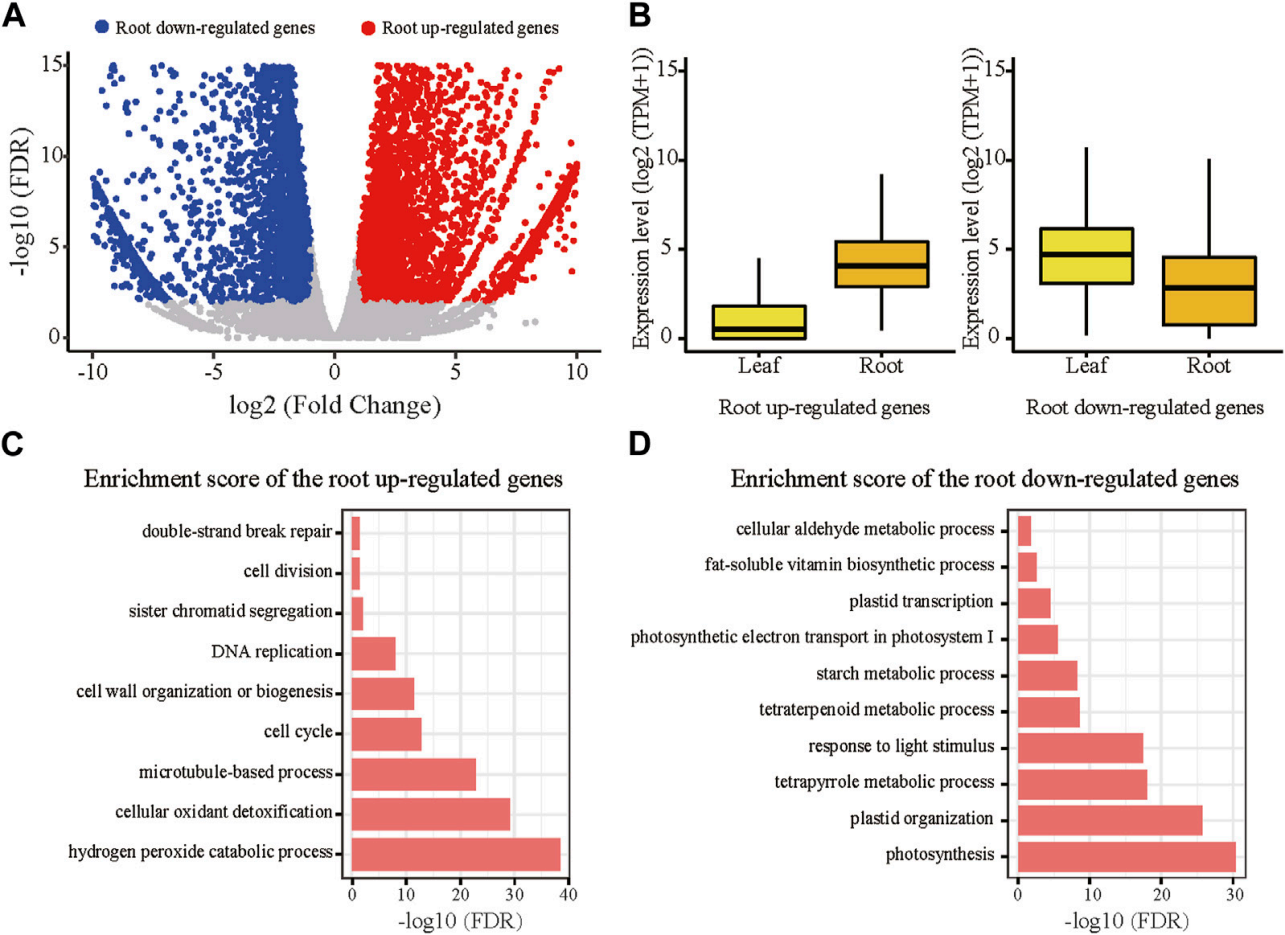
Differentially expressed genes (DEGs) between the root and leaf tissues in the tetraploid line. **(A)** Upregulated genes in root were marked with red color; downregulate genes in root were marked with blue color; non-DEGs were marked with gray color. DEGs were defined with the following criteria: FDR<0.01, fold change >1. **(B)** Expression levels of the root upregulated and downregulated genes in each tissue of the tetraploid line. **(C)** Significantly enriched GO terms of the root upregulated genes. **(D)** Significantly enriched GO terms of the root downregulated genes.

We conducted RNA-seq for both root and leaf tissues of the tetraploid *Ae. tauschii*. The RNA-seq data between biological replicates were highly correlated (*R* ^*2*^ = 0.97, *p* < 2.2e-16) (Supplementary Figure S2A; Supplementary Table S1). In comparison with leaves, a total of 6,136 genes were significantly upregulated in roots, while 5,660 genes were downregulated (FDR<0.01, log2 Fold Change (FC) > 1) (Figure 1A). The root upregulated genes were expressed at low levels in leaves (with median TPM = 0.4), which substantially differed in roots with median TPM of 16.1 (Figure 1B). In contrast, the differences in the expression levels of the root downregulated genes between leaves (with median TPM = 25.2) and roots (with median TPM of 6.2) were relatively minor (Figure 1B). This result indicates that the root upregulated genes might be associated with functions specific to root. Gene Ontology (GO) analysis revealed that the root upregulated genes were significantly enriched in terms related to hydrogen peroxide catabolic process, cell cycle and cell division (Figure 1C). The concentration of hydrogen peroxide in the root differentiation zone and the cell wall of root hairs affect the root elongation and root hair formation (Dunand et al., 2007). In addition, hydrogen peroxide was also found to be involved in response to abiotic stresses in roots (Hernandez et al., 2010). These functions are consistent with the root-specific upregulation of the genes. As expected, the root downregulated genes (that is leaf upregulated genes) were mainly associated with functions of photosynthesis and metabolic process (Figure 1D).

Additionally, we conducted the transcriptome analysis for the diploid line (Supplementary Figure S2B; Supplementary Table S1) and revealed similar results as we found for the tetraploid line (Supplementary Figure S3). We also found 6,863 and 5,621 upregulated and downregulated genes in roots, respectively, compared with leaves. These results collectively suggest that both ploidies tend to be associated with similar tissue-specific expression pattern.

### 3.2 Ploidy-related expression in root and leaf

**FIGURE 2:**
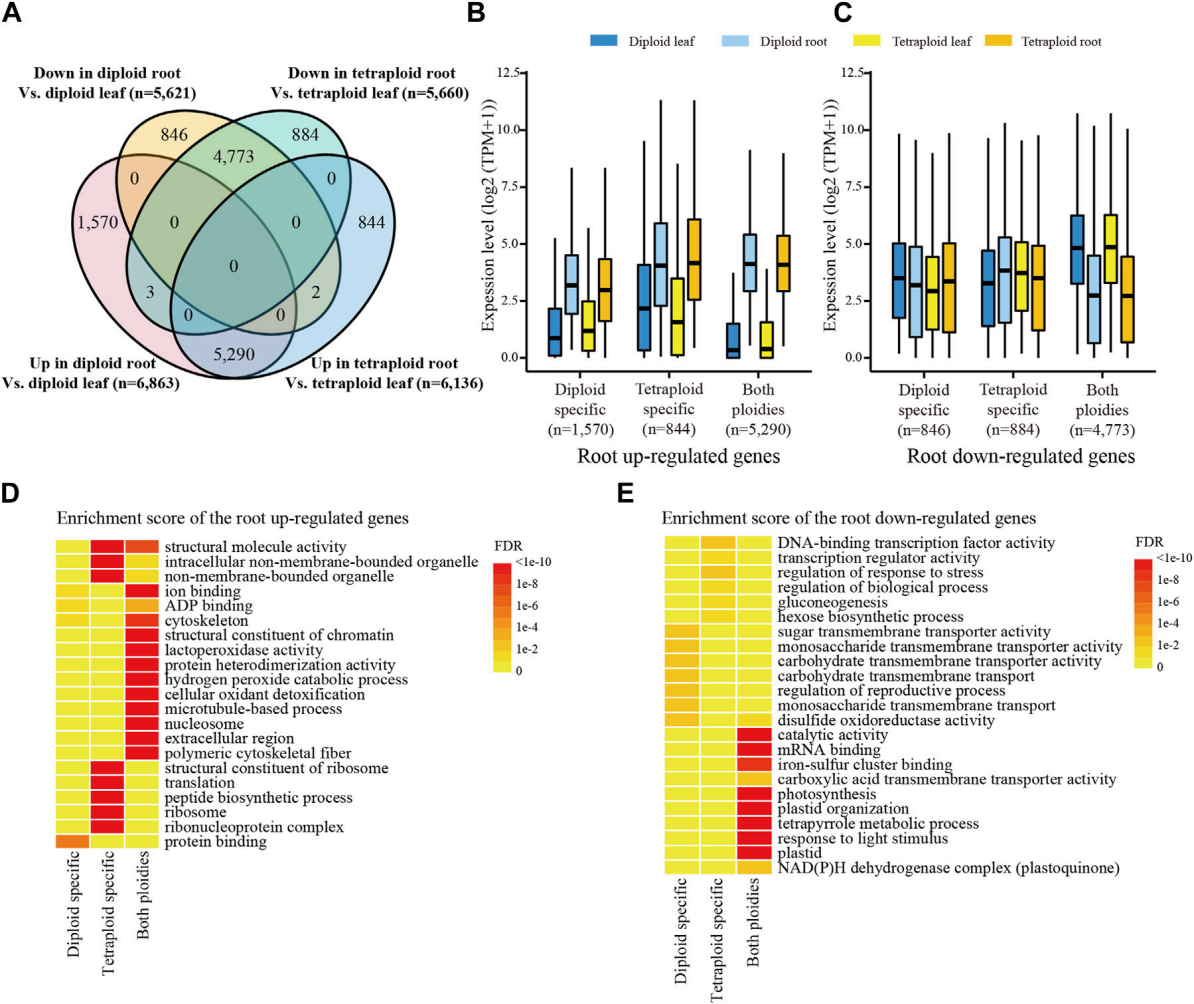
Ploidy-related expression in each tissue. **(A)** Venn diagram of the tissue-DEGs between two ploidies. Each ellipse represents the number of the DEGs between the root and leaf tissues of a given *Ae. tauschii* line. “Up” and “down” represents up- and downregulated genes, respectively. **(B)** The expression levels of the root upregulated genes in each tissue of the diploid and tetraploid lines. **(C)** The expression levels of the root downregulated genes in each tissue of the diploid and tetraploid lines. **(D)** Significantly enriched GO terms of the root upregulated genes. **(E)** Significant enriched GO terms of the root downregulated genes.

Comparison of the tissue-differentially expressed genes (DEGs) between the diploid and tetraploid lines revealed that 77.1% and 86.2% of the root upregulated genes (5,290) displayed the same direction of the tissue-specific differential expression in the diploid and tetraploid lines, respectively (Figure 2A). In contrast, only a small number of the root upregulated genes were specific to the diploid (1,570) and tetraploid lines (884) (Figure 2A). It is noticed that the diploid-specific root upregulated genes (1,570), which were expressed at substantially higher levels in roots than in leaves of the diploid line, also displayed higher expression levels in roots than in leaves of the tetraploid line (Figure 2B). A similar pattern was found for the tetraploid-specific root upregulated genes (884). However, the expression levels of the root downregulated genes were not consistently higher in leaves than in roots (Figure 2C). It is indicated that the root upregulated genes tend to transcribe constantly in both ploidies.

The GO analysis revealed that the majority of the root upregulated genes from both ploidies were enriched in terms associated with cytoskeleton and hydrogen peroxide catabolic process (Figure 2D), which are frequently involved in plant root growth (Dunand et al., 2007; Becker et al., 2014; Takatsuka and Ito, 2020) and response to salt stress (Wang et al., 2011; Chun et al., 2021). The tetraploid-specific root upregulated genes were associated with functions mainly related to ribosome and peptide biosynthetic process (Figure 2D). The peptides, such as the CLE family, are reported to have a role in root apical meristem maintenance, root hair development and lateral root development (Tavormina et al., 2015). Thus, upregulation of the genes related to protein synthesis and its apparatus in the tetraploid line indicates that the tetraploid line might be associated with vigorous roots. The root downregulated genes in both ploidies, however, were mainly associated with functions related to photosynthesis (Figure 2E). The tetraploid-specific root downregulated genes were mainly enriched in stress responses and sugar biosynthesis (Figure 2E). Enhanced stress responses were also found in other polyploid plant species (Dai et al., 2015; Guo et al., 2023), while upregulation of hexose synthesis and gluconeogenesis related genes was observed in sugarcane with high biomass (Wai et al., 2017), suggesting that the tetraploid line might be associated with better tolerance to abiotic stress and biomass production. The diploid-specific root downregulated genes were associated with carbohydrate transmembrane transport activity (Figure 2E), which is involved in enhancing photosynthesis (Ainsworth and Bush, 2011).

### 3.3 Non-TEs sequences within TE-containing genes associated with higher H3K27me3 in the tetraploid line

TEs have been reported to play a role in alternation of gene expression (Gill et al., 2021). To investigate if TEs and their underlying H3K27me3 are potentially associated with the difference in gene expression between two ploidies, we conducted ChIP-seq using antibody against H3K27me3 (Supplementary Table S2). A total of 3,452,972 TEs were identified in the genome. The majority of the TEs were distributed in the intergenic regions, while a noticeable proportion of TEs were located within genic regions (4.5% for Class I and 6.6% for Class II) (Supplementary Figure S4A). The integration of TEs in the genic regions indicates that they might potentially affect the expression of their cognate genes.

**FIGURE 3:**
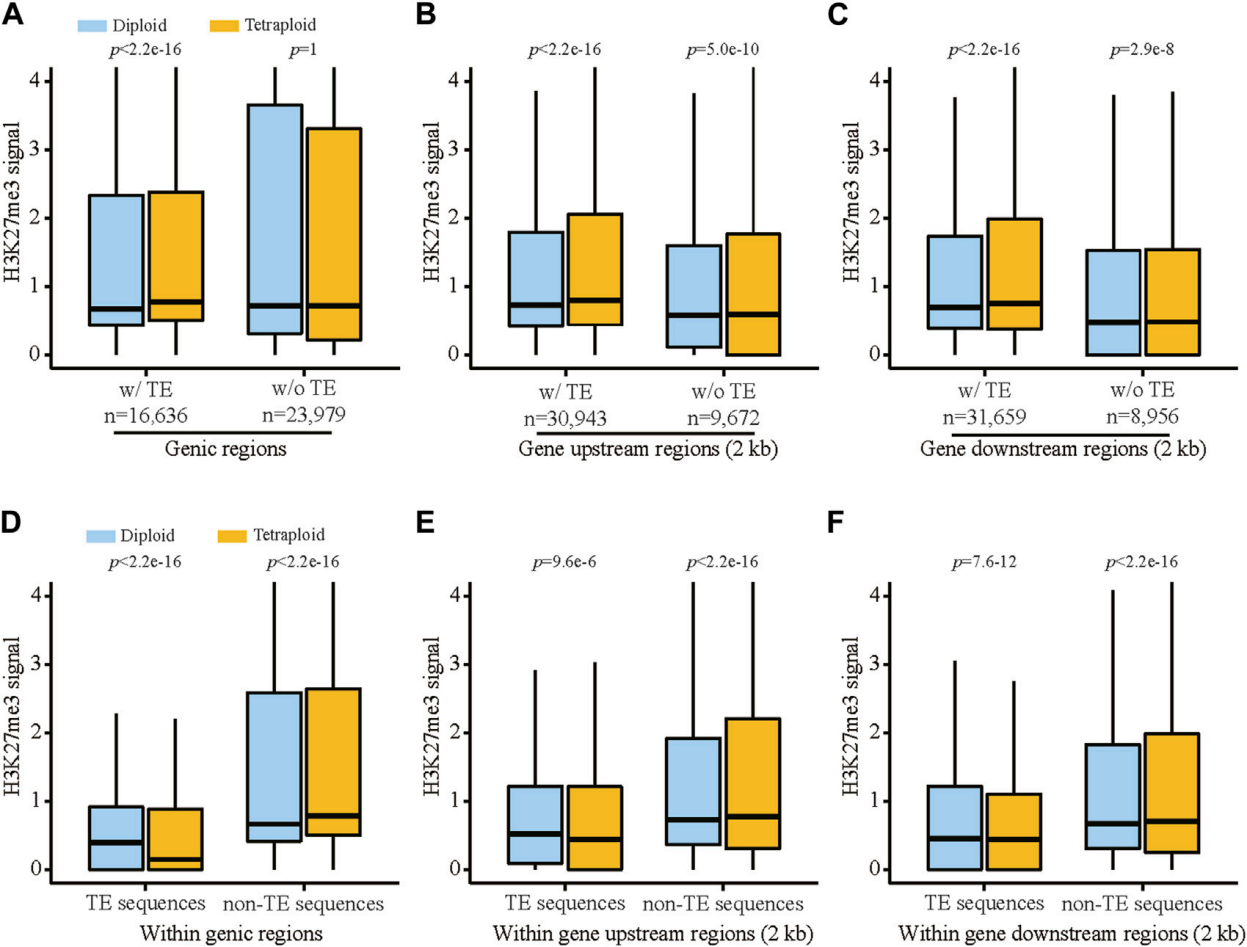
The levels of H3K27me3 associated with TEs and their adjacent non-TE sequences in the vicinity of genes in root tissue of the diploid and tetraploid lines. H3K27me3 signals in genic regions **(A)**, gene 2 kb upstream regions **(B)** and gene 2 kb downstream regions **(C)** associated with and without TEs. H3K27me3 signals in TEs and non-TE sequences located within genic regions **(D)**, gene 2 kb upstream regions **(E)** and gene 2 kb downstream regions **(F)**. Statistical significance was tested using Wilcoxon signed-rank test with paired samples.

The analysis of H3K27me3 revealed that the diploid and tetraploid lines showed generally similar levels of H3K27me3 in TEs located in genic regions as well as the gene upstream regions (2 kb) in leaf tissue (Supplementary Figure S4B). In contrast, in root tissue, the tetraploid line displayed significantly lower levels of H3K27me3 in TEs in the corresponding regions compared with the diploid line (Supplementary Figure S4C). Interestingly, the genomic regions harboring TEs, including the genic regions, 2 kb upstream and 2 kb downstream regions, showed substantially higher levels of H3K27me3 in roots from the tetraploid line than those from the diploid line (Figures 3A–C), suggesting that the flanking regions of TEs within these genomic regions are enriched with H3K27me3 in the tetraploid line. In addition, these genome regions carrying TEs displayed higher levels of H3K27me3 than those without any TEs (Figures 3A–C), indicating TEs might play a role in the deposition of H3K27me3 to the flanking regions of their cognate genes, particularly for the tetraploid line. Dissection of TEs from the flanking regions confirmed that non-TE sequences within the genic regions as well as the upstream and downstream regions were enriched with H3K27me3 in the tetraploid line (Figures 3D–F).

### 3.4 Specific types of TEs might be involved in regulation of ploidy-related gene expression

**FIGURE 4:**
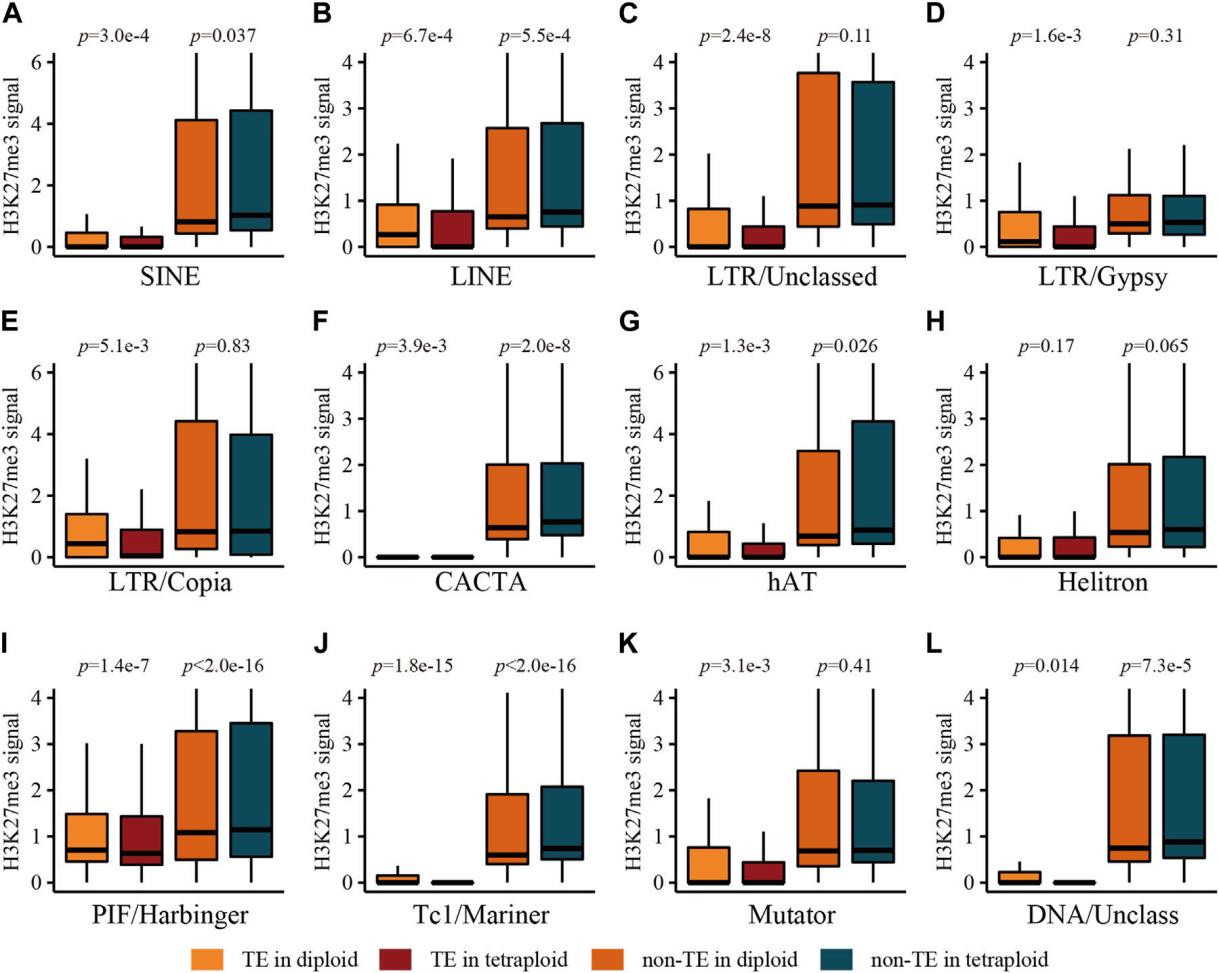
The levels of H3K27me3 associated with main types of TEs and their adjacent non-TE sequences in genic regions in root tissue of the diploid and tetraploid lines. **(A)** SINE, **(B)** LINE, **(C)** LTR/Unclassed, **(D)** LTR/Gypsy, **(E)** LTR/Copia, **(F)** CACTA, **(G)** hAT, **(H)** Helitron, **(I)** PIF/Harbinger, **(J)** Tc1/Mariner, **(K)** Mutator, **(L)** DNA/Unclassed. Statistical significance was tested using Wilcoxon signed-rank test with paired samples.

Further analysis for subclasses of TEs located in genic regions showed that all types of TEs were depleted of H3K27me3 compared with their flanking regions (Figure 4; Supplementary Figure S5). Interestingly, non-TE regions adjacent to LINEs, CACTA, PIF/Harbinger, Tc1/Mariner and unclassed DNA transposon in the tetraploid line were associated with significantly higher levels of H3K27me3 than those in the diploid line (Figure 4), which is coincident with the lower expression levels of their cognate genes in the tetraploid line compared with those in the diploid line (Supplementary Figure S6). The non-TE regions adjacent to the remaining types of TEs displayed similar levels of H3K27me3 between two ploidies (Figure 4), while their corresponding genes were generally expressed at similar levels (Supplementary Figure S6). It is indicated that LINE, CACTA, PIF/Harbinger, Tc1/Mariner and unclassed DNA transposon located within genic regions might be involved in the regulation of the ploidy-related gene expression, possibly through differential enrichment of H3K27me3 in the genic regions.

GO analysis revealed that these TE-associated genes were enriched in various terms of biological processes, including glycolysis, RNA phosphodiester bond hydrolysis, positive regulation of transcription and proteolysis (Supplementary Figure S7). Upregulation of genes associated with glycolysis has been found in roots from multiple genotypes of Banana (*Musa* spp.) (Zorrilla-Fontanesi et al., 2016), presumably involved in higher oxidative respiration. The GO result suggests that the tetraploid line might be associated dynamic energy shift and RNA/protein metabolism. In addition, we extracted the top10 differentially H3K27me3-enriched DEGs (tetraploid line Vs diploid line) carrying these five types of TEs, respectively (Supplementary Table S3). The majority of these genes were likely involved root growth and stress responses in roots, suggesting that the root- and stresses-related differential expression might be regulated with the involvement of H3K27me3 between the tetraploid and diploid lines.

## 4 Discussion

### 4.1 Gene expression might be less affected by ploidy level in roots

WGD usually induces phenotypic changes, typically including enlarged leaves and seeds, increased stem diameter and changed plant architecture. Similar to other plant species (Chen, P. et al., 2020; Guo et al., 2023; Wang et al., 2021; Yu et al., 2010), the tetraploid *Ae. tauschii* line mainly displayed larger leaves and seeds than the diploid line (Zeng et al., 2012). However, the DEGs between two ploidies were limited to a few hundred or less. Interestingly, the ploidy-related DEGs in roots (n = 134, 0.33% of 40,615) were substantially less than those in leaves (n = 528, 1.3% of 40,165). Consistent trend was also reported in previous studies in potato, where the DEGs (n = 27–158) in tubers between the homozygous diploid and tetraploid *Solanum phureja* were only 0.07–0.4% of the total genes (39,400) (Guo et al., 2023), whereas the DEGs (n = 2,652–3,661) in leaves from the other homozygous diploid and tetraploid potato *S. commersonii* accounted for 6.7%–9.3% of the total genes (Fasano et al., 2016). GO analysis for the tetraploid *Ae. tauschii* line upregulated genes revealed that roots were associated with the terms related to conserved function compared with the leaves which were enriched in more specific processes (Supplementary Figure S8). Therefore, it is suggested that the gene expression in *Ae. tauschii* roots might not be affected by ploidy level as much as that in leaves. In addition, similar to potato tubers, roots are non-photosynthesis tissue (Henry et al., 2020), which may be associated with less influences from ambient environment compared with the up-ground tissues.

### 4.2 Specific types of TEs might be involved in ploidy-related gene expression

Immediate WGD from somatic cells likely results in very limited genomic changes in the current generation (Guo et al., 2023). Thus, alternation of gene expression pattern between different ploidies seems to be associated with epigenetic changes. Previous study in rice suggests that WGD increased methylation levels in class II transposable elements, suppressing the genome-wide expression levels of nearby genes (Zhang et al., 2015). Similar evidence was also documented in *Spartina anglica* genome, where TEs were associated with frequent methylation changes (Parisod et al., 2010a), likely leading to transcriptional changes in the vicinity of TEs following allopolyploidization (Parisod et al., 2010b; Parisod et al., 2009). However, studies on WGD-induced epigenetic changes on TEs and their potential effects on adjacent genes are relatively sporadic, while the majority of these studies only focused on DNA methylation (Madlung et al., 2005; Parisod et al., 2009; Zhang et al., 2015). In this study, we attempted to interrogate histone modification features associated with TEs between the diploid and tetraploid *Ae. tauschii* lines. We found genes containing TEs generally displayed higher levels of H3K27me3 in roots, compared with those without TEs, regardless of the ploidy level (Figures 3A–C), indicating that TEs might mediate the deposition of H3K27me3 to their adjacent regions. Interestingly, the TEs located in genic regions were associated with lower levels of H3K27me3 in the tetraploid line compared with those in the diploid line, while the adjacent regions of the TEs within genic regions were associated with higher levels of H3K27me3 in the tetraploid line than those in the diploid line (Figures 3D–F). H3K27me3 and DNA methylation are generally thought to be mutually exclusive (Rougee et al., 2021) and presumably TEs are associated with elevated DNA methylation levels in *Ae. tauschii* (Zhao et al., 2017). Thus, the genic TEs in the tetraploid line might be associated with higher level of DNA methylation than those in the diploid line. Hypermethylation of the TEs following WGD in rice not only suppress the expression of their nearby genes, but also stabilize the genomic structure (Zhang et al., 2015), implying that the regions containing the TEs in the tetraploid *Ae. tauschii* line might be less dynamic in root tissue.

Dissection of TE types revealed that non-TE regions adjacent to five types of TEs in the tetraploid line displayed higher levels of H3K27me3 than those in the diploid line (Figure 4). In addition, the genes containing those types of TEs were generally expressed at lower levels in the tetraploid lines than those in the diploid line (Supplementary Figure S6). The enrichment of H3K27me3 was evidenced in the flanking regions of TEs in *Arabidopsis*, possibly for the formation of heterochromatin and suppression of gene expression (Dong et al., 2012). The differential enrichment of H3K27me3 in these TE-associated genes between the diploid and tetraploid *Ae. tauschii* line may play a role in the ploidy-related gene expression. Collectively, the TEs, including LINE, CACTA, PIF/Harbinger, Tc1/Mariner and unclassed DNA transposon, might be involved in the deposition of H3K27me3 in their adjacent regions for the suppression of the cognate genes. However, this speculation requires further molecular evidence.

## Data Availability

The datasets presented in this study can be found in online repositories. The names of the repository/repositories and accession number(s) can be found in the article/Supplementary Material.

## Supplementary material

SUPPLEMENTARY FIGURE S1 The phenotypes of the diploid and tetraploid *Ae. tauschii* lines. **(A)** The diploid line. **(B)** The tetraploid line. Scale bar = 5 cm.

SUPPLEMENTARY FIGURE S2 Correlation of expression levels between two biological replicates derived from the diploid line **(A)** and the tetraploid line **(B)**, respectively. The Pearson correlation was measured using TPM values of 43,362 annotated *Ae. tauschii* genes.

SUPPLEMENTARY FIGURE S3 DEGs between the root and leaf tissues in the diploid line. **(A)** Up-regulated genes in root were marked with red color; downregulate genes in root were marked with blue color; non-DEGs were marked with gray color. DEGs were defined with the following criteria: FDR < 0.01, fold change >1. **(B)** Expression levels of the root up-regulated and down-regulated genes in each tissue of the diploid line. **(C)** Significantly enriched GO terms of the root up-regulated genes. **(D)** Significantly enriched GO terms of the root down-regulated genes.

SUPPLEMENTARY FIGURE S4 Genomic distribution of TEs identified in the *Ae. tauschii* genome. **(A)** Genomic distribution of TEs relative to the annotated genes. Class I and Class II represent retrotransposons and transposons, respectively. TE was assigned to a genomic feature if more than half length of TEs is overlapped with a genomic feature. Upstream indicates the region 2 kb upstream from a gene. Downstream indicates the region 2 kb downstream from a gene. Intergenic refers to the region 2 kb away from any genes. **(B)** The levels of H3K27me3 associated with TEs located in various genomic regions in leaf tissue. **(C)** The levels of H3K27me3 associated with TEs located in various genomic regions in root tissue. Statistical significance was tested using Wilcoxon signed-rank test with paired samples.

SUPPLEMENTARY FIGURE S5 The profile of H3K27me3 associated with TEs located in genic regions. **(A)** SINE, **(B)** LINE, **(C)** LTR/Unclassed, **(D)** LTR/Gypsy, **(E)** LTR/Copia, **(F)** CACTA, **(G)** hAT, **(H)** Helitron, **(I)** PIF/Harbinger, **(J)** Tc1/Mariner, **(K)** Mutator, **(L)** DNA/Unclassed. Genes flanking regions (±1 kb) were analyzed in 50 bins.

SUPPLEMENTARY FIGURE S6 Comparison of the expression levels of genes containing specific types of TEs between the diploid and tetraploid lines. The expression data was derived from the root tissue. Statistical significance was tested using Wilcoxon signed-rank test with paired samples.

SUPPLEMENTARY FIGURE S7 Significantly enriched GO terms of the genes containing specific types of TEs.

SUPPLEMENTARY FIGURE S8 Significantly enriched GO terms of the upregulated genes in the tetraploid line. Leaf represents the upregulated genes in leaf tissue of the tetraploid line in comparison with the leaf tissue of the diploid line. Root represents the upregulated genes in root tissue of the tetraploid line in comparison with the root tissue of the diploid line.
