# Supplementary figures and images for: Whole genome doubling-induced the enrichment of H3K27me3 in genes carrying specific TEs in Aegilops tauschii
Source: Front Genet. 2023 Jul 25;14:1241201. doi: 10.3389/fgene.2023.1241201 (PMC10407559; doi:10.3389/fgene.2023.1241201)

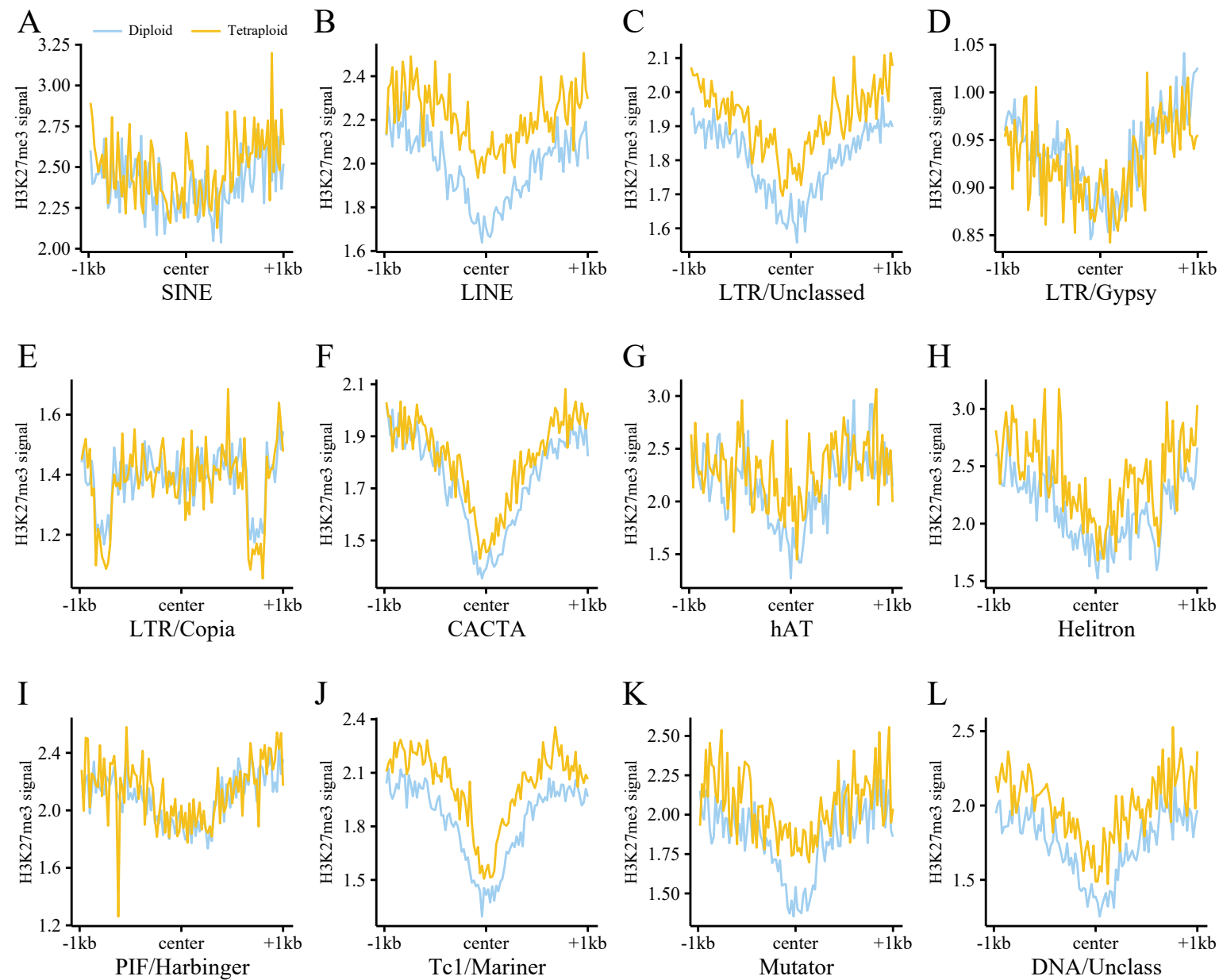

Figure S5

Supplement: Supplementary file 1 [file Image5.PDF]

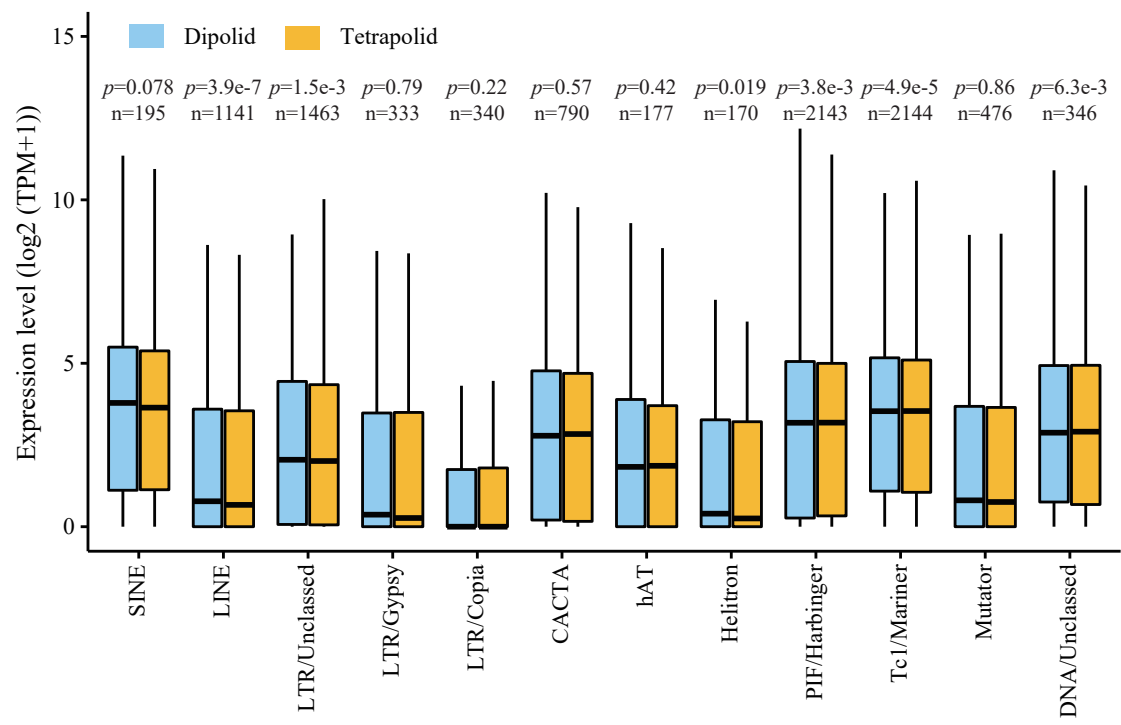

Figure S6

Supplement: Supplementary file 3 [file Image6.PDF]

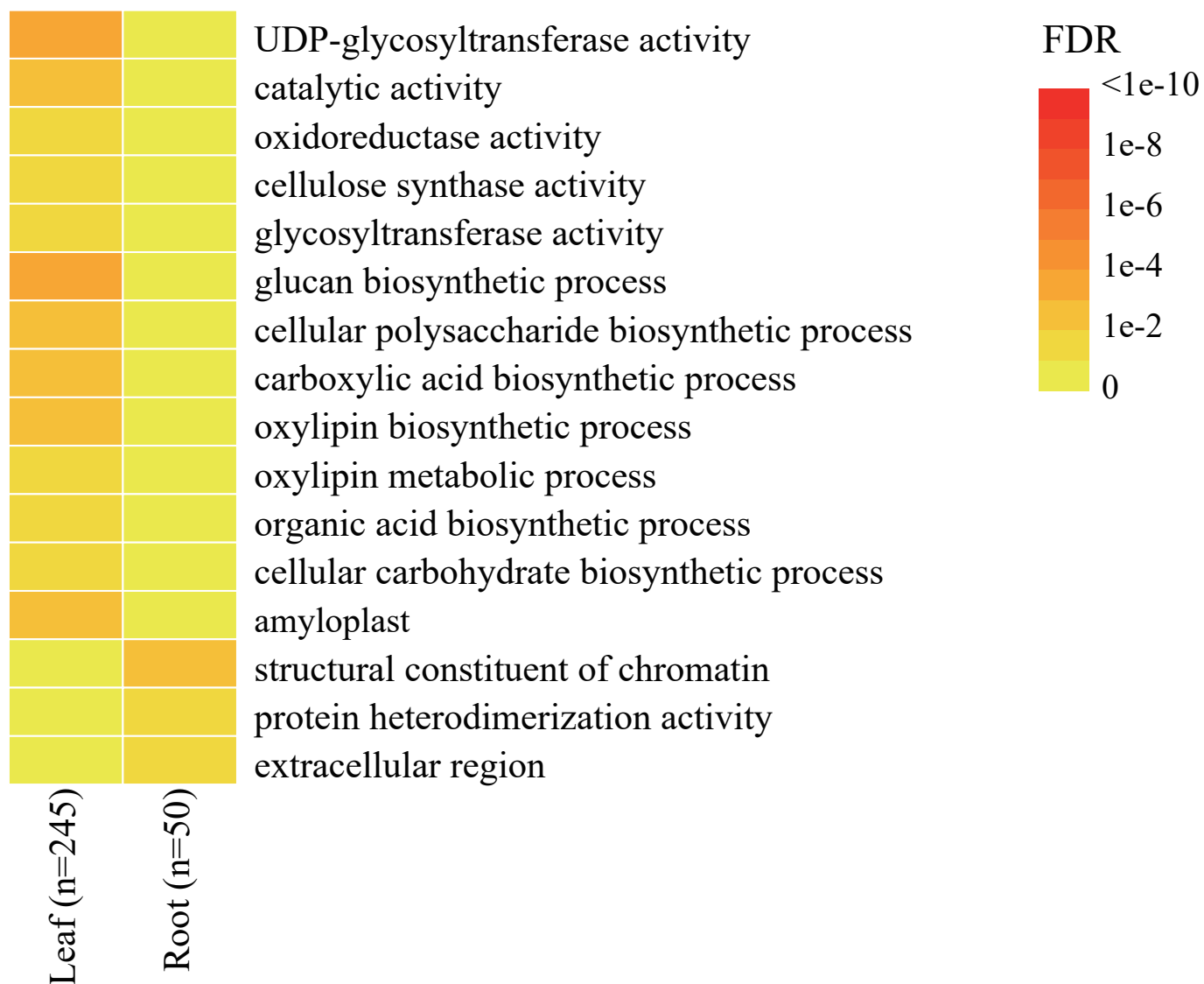

Figure S8

Supplement: Supplementary file 4 [file Image8.PDF]

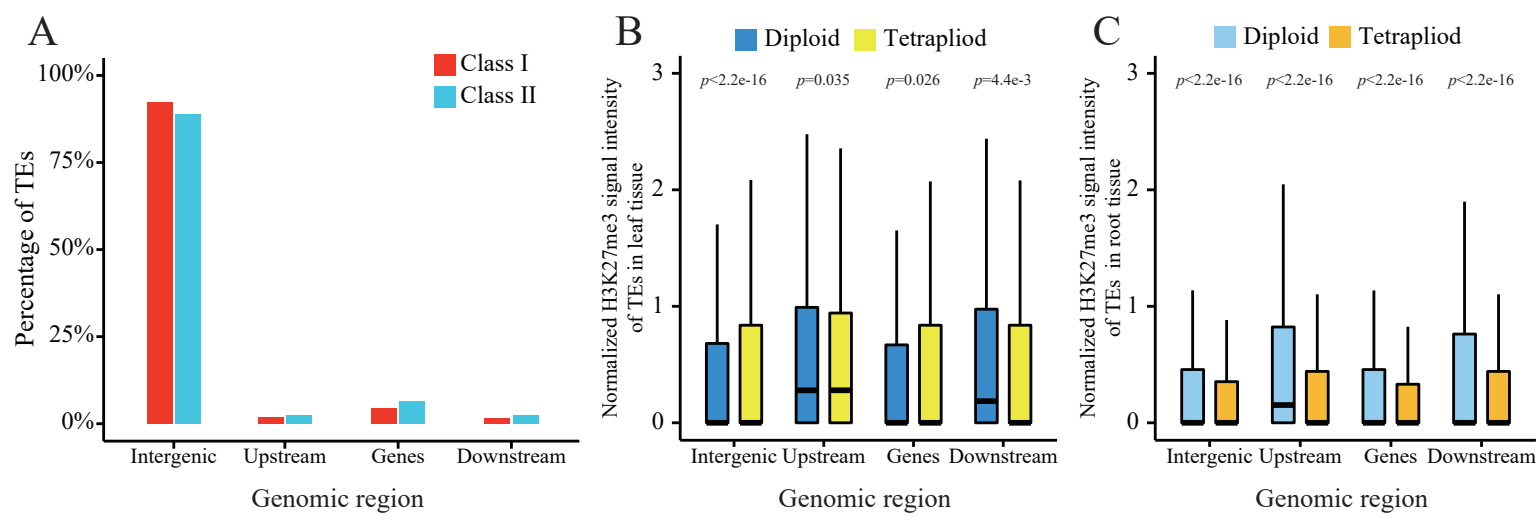

Figure S4

Supplement: Supplementary file 5 [file Image4.PDF]

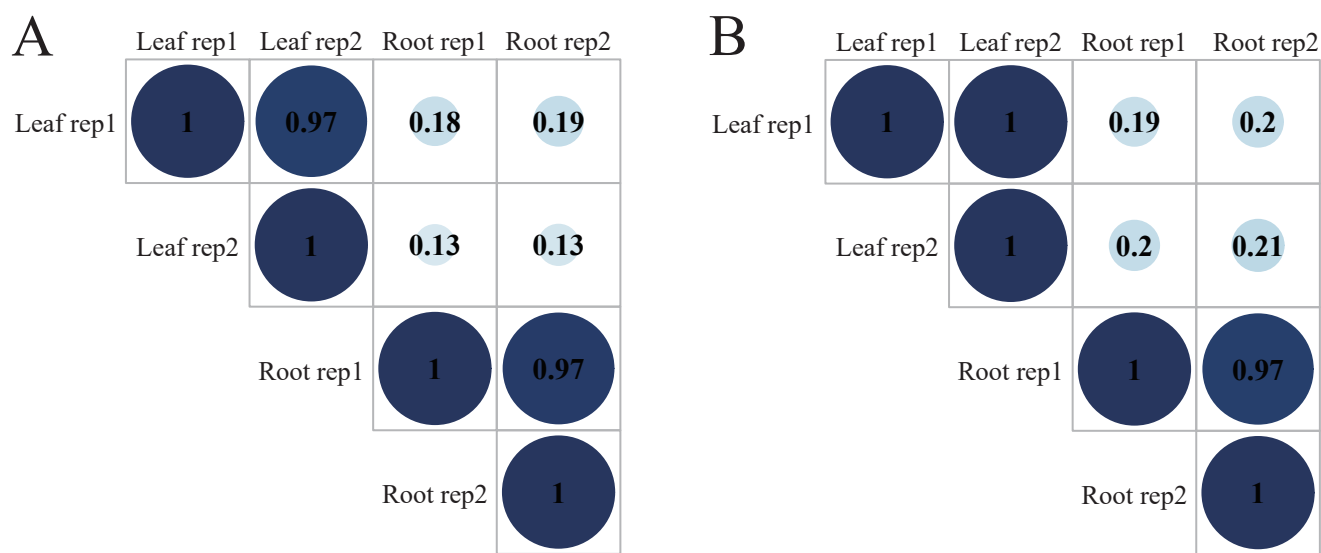

The tetraploid line

The diploid line

Figure S2

Supplement: Supplementary file 6 [file Image2.PDF]

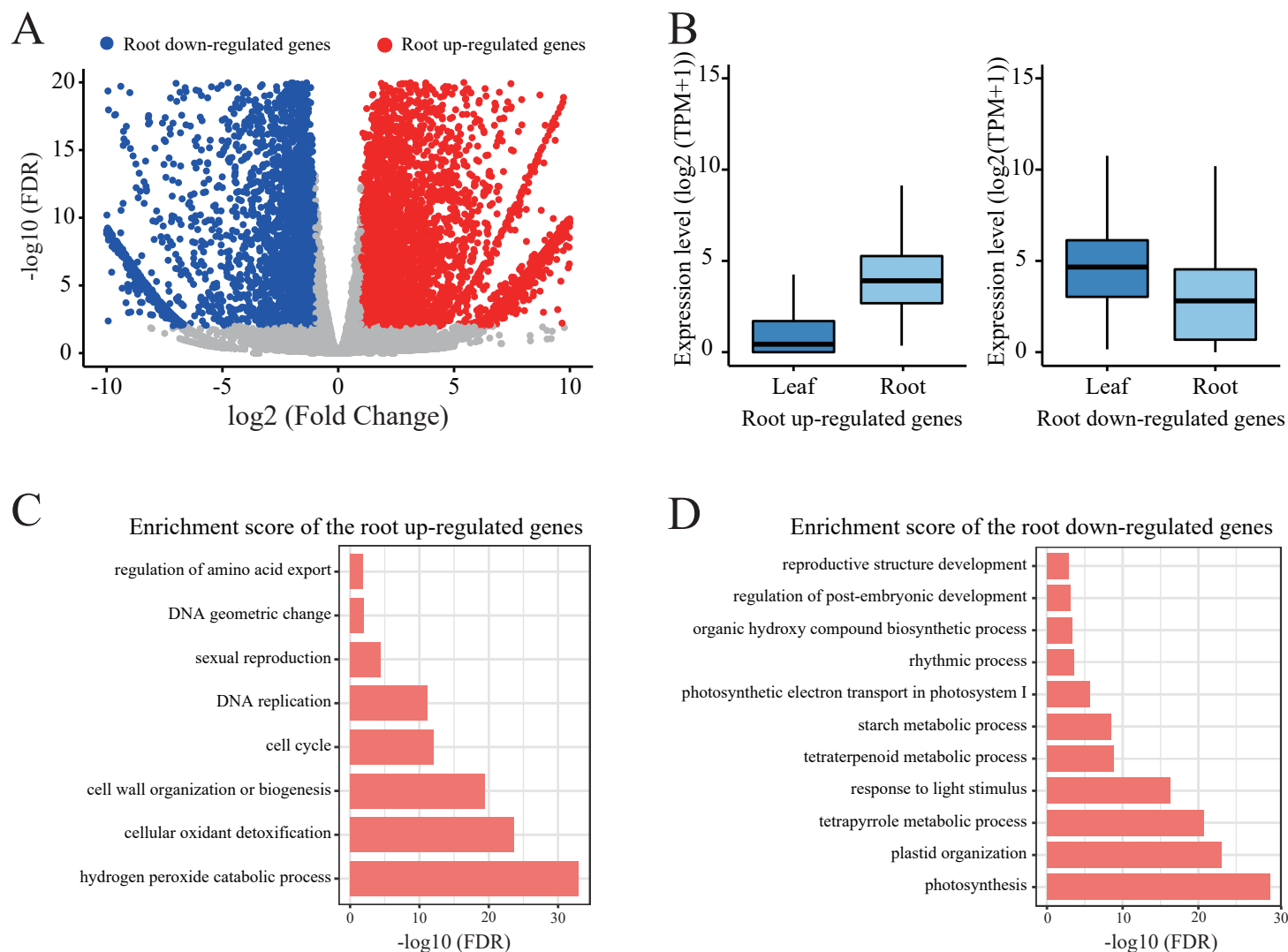

Figure S3

Supplement: Supplementary file 7 [file Image3.PDF]

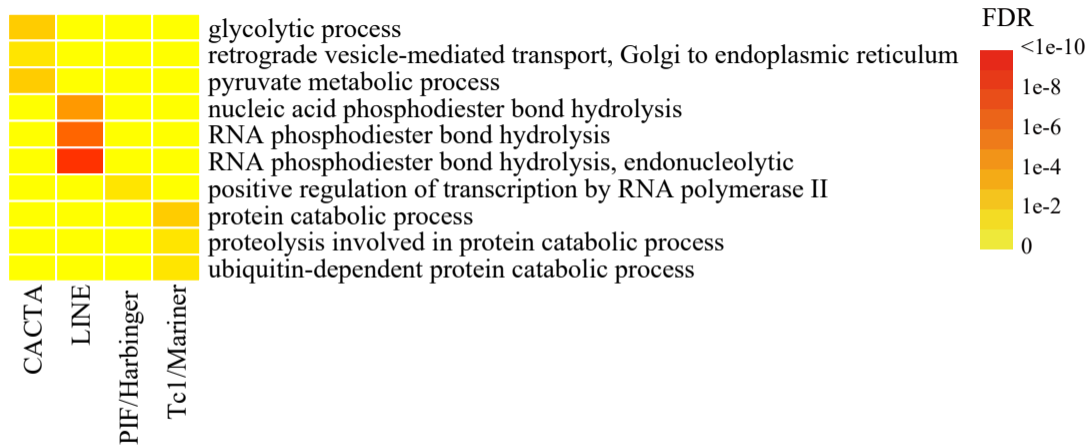

Figure S7

Supplement: Supplementary file 8 [file Image7.PDF]

A

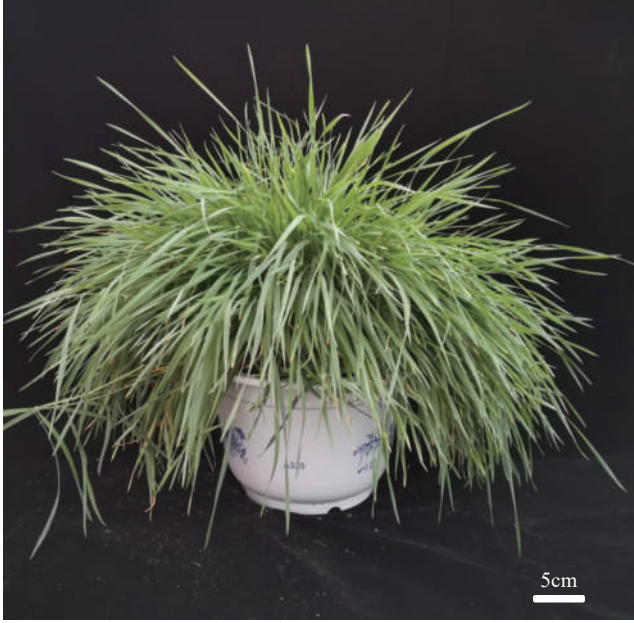

B

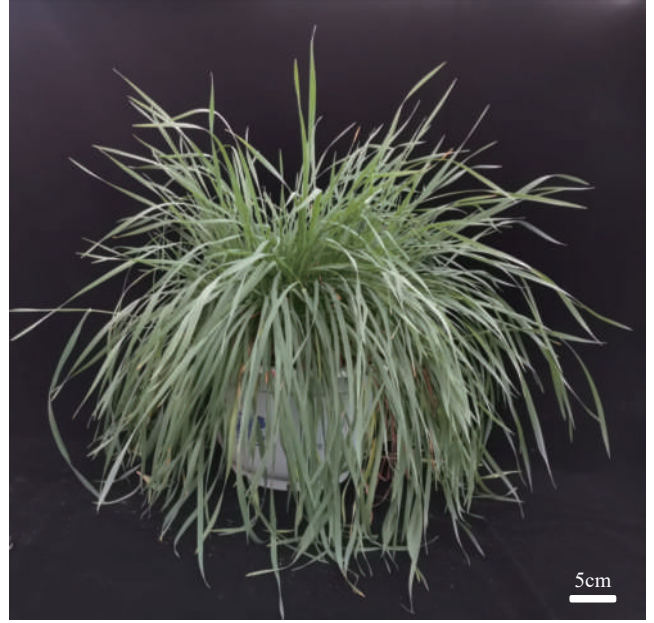

Figure S1

Supplement: Supplementary file 9 [file Image1.PDF]
