# Supplementary material for: Whole genome doubling-induced the enrichment of H3K27me3 in genes carrying specific TEs in Aegilops tauschii
Source: Front Genet. 2023 Jul 25;14:1241201. doi: 10.3389/fgene.2023.1241201 (PMC10407559; doi:10.3389/fgene.2023.1241201)
Supplement: Supplementary file 2 [file Table1.DOCX]

| **Table S1. RNA-seq data generated from leaves and roots of the diploid and tetraploid *Aegilops tauschii* Coss. lines.** | | |
| --- | --- | --- |
| **Ploidy** | **Tissue** | **Uniquely mapped PE reads #** |
| Diploid line | Leaf Rep1 | 20,264,065 |
| Diploid line | Leaf Rep2 | 17,968,266 |
| Diploid line | Root Rep1 | 19,488,480 |
| Diploid line | Root Rep2 | 17,467,047 |
| Tetraploid line | Leaf Rep1 | 20,077,320 |
| Tetraploid line | Leaf Rep2 | 20,774,424 |
| Tetraploid line | Root Rep1 | 17,770,830 |
| Tetraploid line | Root Rep2 | 16,783,389 |

| **Table S2. ChIP-seq data generated from leaves and roots of the diploid and tetraploid *Aegilops tauschii* Coss. lines.** | | | |
| --- | --- | --- | --- |
| **Ploidy** | **Tissue** | **Antibody** | **Uniquely mapped PE reads #** |
| Diploid line | Leaf | H3K27me3 | 34,103,553 |
| Diploid line | Leaf | IgG | 18,989,816 |
| Diploid line | Root | H3K27me3 | 34,684,705 |
| Diploid line | Root | IgG | 15,887,007 |
| Tetraploid line | Leaf | H3K27me3 | 22,776,949 |
| Tetraploid line | Leaf | IgG | 19,091,500 |
| Tetraploid line | Root | H3K27me3 | 20,710,960 |
| Tetraploid line | Root | IgG | 9,155,619 |

| **Table S3. The information of the Top10 H3K27me3 enriched and tetraploid line up-regulated genes carrying five types of TEs in the root.** | | | | |
| --- | --- | --- | --- | --- |
| **TE type** | **Protein name** | **Gene ID** | **Function related to root** | **Reference** |
| LINE | Unknown | AET7Gv20037700 | NA |  |
|  | Anthocyanidin 5,3-O-glucosyltransferase | AET4Gv20881300 | Abiotic stress response | (Hirotani et al., 2000) |
|  | CHLORORESPIRATORY REDUCTION 6, chloroplastic | AET6Gv20124800 | NA |  |
|  | Putative spermidine synthase | AET7Gv20256600 | Abiotic stress response | (Kasukabe et al., 2006) |
|  | Unknown | AET1Gv20806900 | NA |  |
|  | Unknown | AET5Gv20599600 | NA |  |
|  | Unknown | AET5Gv21233800 | NA |  |
|  | Casein kinase I-2-like protein | AET5Gv21000700 | Root growth and abiotic stress response | (Mulekar et al., 2012) |
|  | Unknown | AET3Gv20190300 | NA |  |
|  | Dehydration-responsive element-binding protein 1A | AET5Gv20720000 | Abiotic stress response | (Hong and Kim, 2005) |
| CACTA | UPF0481 protein | AET3Gv20014700 | NA |  |
|  | Disease resistance protein RPM1 | AET7Gv20087200 | Biotic stress response | (Nie and Ji, 2019) |
|  | Ethylene-responsive transcription factor WRI1 | AET5Gv20535200 | Primary root growth | (Zhang et al., 2019) |
|  | F-box domain-containing protein | AET5Gv21169800 | Abiotic stress response | (Zhou et al., 2014) |
|  | DUF309 domain-containing protein | AET4Gv20267500 | Root crown | (Zhou et al., 2017) |
|  | Putative acetyltransferase | AET1Gv20818100 | NA | NA |
|  | protein-serine/threonine phosphatase | AET2Gv21006500 | Abiotic stress response | (Lin et al., 2009) |
|  | Subtilisin-like protease | AET5Gv20113200 | Abiotic stress response | (Golldack et al., 2003) |
|  | Unknown | AET5Gv20670800 | NA |  |
|  | Unknown | AET4Gv20489300 | NA |  |
| PIF/Harbinger | Unknown | AET2Gv21288600 | NA |  |
|  | CASP-like protein | AET5Gv20931700 | Abiotic stress response | (Yang et al., 2015) |
|  | Disease resistance protein RPM1 | AET2Gv20074900 | Biotic stress response | (Nie and Ji, 2019) |
|  | Unknown | AET5Gv20611600 | NA |  |
|  | rRNA N-glycosylase | AET6Gv20025500 | Biotic stress response | (Landi et al., 2022) |
|  | Unknown | AET1Gv20082100 | NA |  |
|  | Dirigent protein | AET4Gv20633700 | Biotic and abiotic stresses response | (Paniagua et al., 2017) |
|  | Unknown | AET2Gv21203800 | NA |  |
|  | Cytokinin-N-glucosyltransferase 1 | AET3Gv21186800 | Responses of exogenous cytokinins in main and lateral root | (Wang et al., 2011) |
|  | Unknown | AET1Gv20766300 | NA |  |
| TC1/Mariner | Cation/calcium exchanger 3 | AET2Gv20230200 | Abiotic stress response | (Yang et al., 2021) |
|  | Unknown | AET4Gv20816200 | NA |  |
|  | O-methyltransferase ZRP4 | AET7Gv20104700 | Low levels in root meristematic regions, high levels in the region of cell maturation | (Held et al., 1993) |
|  | Putative inactive receptor kinase | AET5Gv21169300 | NA |  |
|  | protein-serine/threonine phosphatase | AET1Gv20978800 | Abiotic stress response | (Lin et al., 2009) |
|  | O-methyltransferase ZRP4 | AET3Gv20887900 | Low levels in root meristematic regions, high levels in the region of cell maturation | (Held et al., 1993) |
|  | DC1 domain-containing protein | AET2Gv21085700 | Abiotic stress response | (Li et al., 2010) |
|  | Cytochrome P450 71A9 | AET4Gv20881400 | NA |  |
|  | Unknown | AET7Gv21299300 | NA |  |
|  | Cytochrome P450 71D7 | AET3Gv20170200 | NA |  |
| unclassed DNA transposon | Unknown | AET3Gv20674400 | NA |  |
|  | Unknown | AET5Gv20989400 | NA |  |
|  | ABC transporter D family member 1 | AET3Gv21212700 | Abiotic stress response | (Kang et al., 2010) |
|  | Dof zinc finger protein | AET4Gv20696000 | Involved in alternation of carbohydrate metabolism in roots | (Tanaka et al., 2009) |
|  | Aquaporin NIP1-3 | AET5Gv20789900 | Water usage | (Pandey et al., 2013) |
|  | Glycosyltransferase | AET2Gv20130100 | Abiotic stress response | (Ahrazem et al., 2015) |
|  | Unknown | AET7Gv21058300 | NA |  |
|  | Unknown | AET5Gv20028100 | NA |  |
|  | Ankyrin-1 | AET5Gv20025500 | NA |  |
|  | Unknown | AET6Gv20143700 | NA |  |

**References**

Ahrazem, O., Rubio-Moraga, A., Trapero-Mozos, A., Climent, M.F., Gomez-Cadenas, A., Gomez-Gomez, L., 2015. Ectopic expression of a stress-inducible glycosyltransferase from saffron enhances salt and oxidative stress tolerance in Arabidopsis while alters anchor root formation. Plant Sci 234, 60-73. https://doi.org/10.1016/j.plantsci.2015.02.004.

Golldack, D., Vera, P., Dietz, K.J., 2003. Expression of subtilisin-like serine proteases in Arabidopsis thaliana is cell-specific and responds to jasmonic acid and heavy metals with developmental differences. Physiol Plant 118(1), 64-73. https://doi.org/10.1034/j.1399-3054.2003.00087.x.

Held, B.M., Wang, H., John, I., Wurtele, E.S., Colbert, J.T., 1993. An mRNA putatively coding for an O-methyltransferase accumulates preferentially in maize roots and is located predominantly in the region of the endodermis. Plant Physiol 102(3), 1001-1008. https://doi.org/10.1104/pp.102.3.1001.

Hirotani, M., Kuroda, R., Suzuki, H., Yoshikawa, T., 2000. Cloning and expression of UDP-glucose: flavonoid 7-O-glucosyltransferase from hairy root cultures of Scutellaria baicalensis. Planta 210(6), 1006-1013. https://doi.org/10.1007/PL00008158.

Hong, J.P., Kim, W.T., 2005. Isolation and functional characterization of the Ca-DREBLP1 gene encoding a dehydration-responsive element binding-factor-like protein 1 in hot pepper (Capsicum annuum L. cv. Pukang). Planta 220(6), 875-888. https://doi.org/10.1007/s00425-004-1412-5.

Kang, J., Hwang, J.U., Lee, M., Kim, Y.Y., Assmann, S.M., Martinoia, E., Lee, Y., 2010. PDR-type ABC transporter mediates cellular uptake of the phytohormone abscisic acid. Proc Natl Acad Sci U S A 107(5), 2355-2360. https://doi.org/10.1073/pnas.0909222107.

Kasukabe, Y., He, L., Watakabe, Y., Otani, M., Shimada, T., Tachibana, S., 2006. Improvement of environmental stress tolerance of sweet potato by introduction of genes for spermidine synthase. Plant Biotechnology 23(1), 75-83.

Landi, N., Ragucci, S., Citores, L., Clemente, A., Hussain, H.Z.F., Iglesias, R., Ferreras, J.M., Di Maro, A., 2022. Isolation, Characterization and Biological Action of Type-1 Ribosome-Inactivating Proteins from Tissues of Salsola soda L. Toxins (Basel) 14(8). https://doi.org/10.3390/toxins14080566.

Li, C., Lv, J., Zhao, X., Ai, X., Zhu, X., Wang, M., Zhao, S., Xia, G., 2010. TaCHP: a wheat zinc finger protein gene down-regulated by abscisic acid and salinity stress plays a positive role in stress tolerance. Plant Physiol 154(1), 211-221. https://doi.org/10.1104/pp.110.161182.

Lin, C.W., Lin, C.Y., Chang, C.C., Lee, R.H., Tsai, T.M., Chen, P.Y., Chi, W.C., Huang, H.J., 2009. Early signalling pathways in rice roots under vanadate stress. Plant Physiol Biochem 47(5), 369-376. https://doi.org/10.1016/j.plaphy.2009.01.005.

Mulekar, J.J., Bu, Q., Chen, F., Huq, E., 2012. Casein kinase II alpha subunits affect multiple developmental and stress-responsive pathways in Arabidopsis. Plant J 69(2), 343-354. https://doi.org/10.1111/j.1365-313X.2011.04794.x.

Nie, Y., Ji, W., 2019. Cloning and characterization of disease resistance protein RPM1 genes against powdery mildew in wheat line N9134. Cereal Research Communications 47, 473-483.

Pandey, B., Sharma, P., Pandey, D.M., Sharma, I., Chatrath, R., 2013. Identification of new aquaporin genes and single nucleotide polymorphism in bread wheat. Evol Bioinform Online 9, 437-452. https://doi.org/10.4137/EBO.S12568.

Paniagua, C., Bilkova, A., Jackson, P., Dabravolski, S., Riber, W., Didi, V., Houser, J., Gigli-Bisceglia, N., Wimmerova, M., Budinska, E., Hamann, T., Hejatko, J., 2017. Dirigent proteins in plants: modulating cell wall metabolism during abiotic and biotic stress exposure. J Exp Bot 68(13), 3287-3301. https://doi.org/10.1093/jxb/erx141.

Tanaka, M., Takahata, Y., Nakayama, H., Nakatani, M., Tahara, M., 2009. Altered carbohydrate metabolism in the storage roots of sweetpotato plants overexpressing the SRF1 gene, which encodes a Dof zinc finger transcription factor. Planta 230, 737-746.

Wang, J., Ma, X.M., Kojima, M., Sakakibara, H., Hou, B.K., 2011. N-glucosyltransferase UGT76C2 is involved in cytokinin homeostasis and cytokinin response in Arabidopsis thaliana. Plant Cell Physiol 52(12), 2200-2213. https://doi.org/10.1093/pcp/pcr152.

Yang, J., Ding, C., Xu, B., Chen, C., Narsai, R., Whelan, J., Hu, Z., Zhang, M., 2015. A Casparian strip domain-like gene, CASPL, negatively alters growth and cold tolerance. Sci Rep 5, 14299. https://doi.org/10.1038/srep14299.

Yang, J., Li, W., Guo, X., Chen, P., Cheng, Y., Mao, K., Ma, F., 2021. Cation/Ca(2+) Exchanger 1 (MdCCX1), a Plasma Membrane-Localized Na(+) Transporter, Enhances Plant Salt Tolerance by Inhibiting Excessive Accumulation of Na(+) and Reactive Oxygen Species. Front Plant Sci 12, 746189. https://doi.org/10.3389/fpls.2021.746189.

Zhang, Q., Sun, R., Zheng, Y., Yuan, Y., Li, D., 2019. Isolation and characterization of the EgWRI1 promoter from oil palm (Elaeis guineensis Jacq.) and its response to environmental stress and ethylene. PLoS One 14(12), e0225115. https://doi.org/10.1371/journal.pone.0225115.

Zhou, S., Jiang, W., Long, F., Cheng, S., Yang, W., Zhao, Y., Zhou, D.X., 2017. Rice Homeodomain Protein WOX11 Recruits a Histone Acetyltransferase Complex to Establish Programs of Cell Proliferation of Crown Root Meristem. Plant Cell 29(5), 1088-1104. https://doi.org/10.1105/tpc.16.00908.

Zhou, S., Sun, X., Yin, S., Kong, X., Zhou, S., Xu, Y., Luo, Y., Wang, W., 2014. The role of the F-box gene TaFBA1 from wheat (Triticum aestivum L.) in drought tolerance. Plant Physiol Biochem 84, 213-223. https://doi.org/10.1016/j.plaphy.2014.09.017.
